# Effect of Phytoplasma Associated with Sesame Phyllody on Ultrastructural Modification, Physio-Biochemical Traits, Productivity and Oil Quality

**DOI:** 10.3390/plants11040477

**Published:** 2022-02-10

**Authors:** Eman A. Ahmed, Amro A. Farrag, Ahmed A. Kheder, Ahmed Shaaban

**Affiliations:** 1 Virus and Phytoplasma Research Department, Plant Pathology Research Institute, Agricultural Research Center, Giza 12619, Egypt; mnassar9977@gmail.com (E.A.A.); pl_virology@yahoo.com (A.A.F.); ahmed.zakaria@arc.sci.eg (A.A.K.); 2 Agronomy Department, Faculty of Agriculture, Fayoum University, Fayoum 63514, Egypt

**Keywords:** phytoplasma, sesame phyllody, 16SII phytoplasma group, physio-biochemical responses, oil yield and fatty acid profile, oil properties

## Abstract

Phytoplasmas are obligate cell-wall-less plant pathogenic bacteria that infect many economically important crops, causing considerable yield losses worldwide. Very little information is known about phytoplasma–host plant interaction mechanisms and their influence on sesame yield and oil quality. Therefore, our aim was to explore the ultrastructural and agro-physio-biochemical responses of sesame plants and their effects on sesame productivity and oil quality in response to phytoplasma infection. Sesame leaf samples exhibiting phyllody symptoms were collected from three experimental fields during the 2021 growing season. Phytoplasma was successfully detected by nested- polymerase chain reaction (PCR) assays using the universal primer pairs P1/P7 and R16F2n/R16R2, and the product of approximately 1200 bp was amplified. The amplified product of 16S rRNA was sequenced and compared with other available phytoplasma’s 16S rRNA in the GenBank database. Phylogenetic analysis revealed that our Egyptian isolate under accession number MW945416 is closely related to the 16SrII group and showed close (99.7%) identity with MH011394 and L33765.1, which were isolated from Egypt and the USA, respectively. The microscopic examination of phytoplasma-infected plants revealed an observable deterioration in tissue and cell ultrastructure. The primary and secondary metabolites considerably increased in infected plants compared with healthy ones. Moreover, phytoplasma-infected plants showed drastically reduced water content, chlorophyll content, growth, and yield components, resulting in 37.9% and 42.5% reductions in seed and oil yield, respectively. The peroxide value of the infected plant’s oil was 43.2% higher than that of healthy ones, suggesting a short shelf-life. Our findings will provide a better understanding of the phyllody disease pathosystem, helping us to develop effective strategies for overcoming such diseases.

## 1. Introduction

Sesame, scientifically known as *Sesamum indicum* (L.), is a diploid 2n = 26 annual plant of the *Pedaliaceae* family and is typically grown in global tropic and subtropic areas, especially in Africa and Asia [1]. As an ancient and important oily seed crop, sesame is cultivated mainly for its valuable edible oil used for human cooking oil and animal feeds, besides its positive human health benefits [2]. Sesame seeds contain 32.8–62.7% vegetable oils rich in monounsaturated essential fatty acids (~85%), mainly oleic and linoleic [3]. Sesame oils are rich in lipid-soluble lignans (mainly sesamol, sesamin, and sesamolin), which protect it from oxidative-induced rancidity, prolonging its shelf-life [4]. Sesame seeds are an excellent source of proteins (14.1–29.5%), carbohydrates (4.5–20.5%), fiber (2.7–6.7%), vitamins (E; α-tocopherol and B1; thiamine), minerals, and phytosterols [4,5]. The global cultivated acreage in 2019 was ~12.82 million hectares, producing ~64.5 million tons of sesame seeds that supplied ~12% of the gross world consumption of vegetable oils [6]. In Egypt, the total cultivated sesame acreage in 2019 was ~33,000 hectares, producing ~41,000 tons, with an average oilseed yield of about 1.2 t hectare^−1^ (ha^−1^) [7]. Because of dramatic population growth, Egypt is experiencing a severe lack of edible oils, as the domestic production of vegetable oils is ~0.25 million tons compared to the ~1.7 million t demanded local consumption. This signifies a considerable gap (85.3%) between consumption and production, which has resulted in importing edible oils from outside Egypt to fulfill local market requirements [8]. To bridge this gap, it is essential to enhance sesame production. However, many sesame cropland areas, particularly in arid and semi-arid areas of the world (such as Egypt), face various environmental abiotic (e.g., salinity, drought, heavy metals, low soil fertility, etc.) and biotic (e.g., diseases, pests, etc.) stresses. In the last decade, considerable yield losses in many crops, including sesame, were recorded due to various plant diseases acting as biotic stresses.

Among these serious diseases, phyllody, a phytoplasma-related disease, is considered one of the main hindrances in many areas, especially in Asia and Africa, causing significant losses in sesame yield quantity and quality [9,10]. In India, as one of the top ten sesame-producing countries globally, the incidence of sesame phyllody disease reaches from 10 to 100% in some croplands [11]. Akhtar et al. [12] and Salehi et al. [13] reported that sesame phyllody has been a destructive disease and may cause 80% seed yield loss. According to Vasudeva and Sahambi [14], phytoplasma-associated phyllody disease was first detected in Pakistan in 1908. For a long time, phyllody has been considered a viral-associated plant pathology [15]. However, many researchers [10,11,12] have reported phyllody disease associated with phytoplasma infection.

Phytoplasmas are wall-less prokaryotes, taxonomically belonging to the class *Mollicutes*; they usually colonize the nutrient-rich phloem tissue and appear as very small (400 to 900 nm diameter) spherical to oval or elongated cells in sieve elements (SEs), with a tiny (680 to 1600 kb) genome size. Phytoplasma is transmitted among plants via diseased plant materials in vegetatively propagated crops [16], and phloem-feeding insects (mainly leafhoppers, planthoppers, and psyllids) and dodder are considered the prime means for infecting healthy plants and disseminating infection [17,18]. Phytoplasmas have been detected as pathogens in sesame plants in many parts of the world. As an obligate plant pathogen, the phytoplasma’s pathogenicity in sesame involves various symptoms, as previously described by Youssef et al. [9], Akhtar et al. [19], and El-Banna et al. [20]. Phytoplasmas harm infected plants by affecting various physio-biochemical and metabolic processes [21] and altering genetic expression [22], with the biosynthesis and accumulation of carbohydrates being the most highly affected processes [23]. Phytoplasma-induced harms also include stomatal closure, photosynthetic impairment due to declining leaf area, and photosynthetic pigments, leading to limited transport of photo-assimilates to sink organs, causing yield and quality weakness [21,24,25]. Due to the difficulty of culturing on artificial media under in vitro conditions, the phytoplasma’s detection and identification methods and ecological studies are still restricted. Light microscopy and transmission electron microscopy have been carried out to detect phytoplasmas [18,26,27]. Over the last ten years, molecular DNA-based techniques such as polymerase chain reaction (PCR) and sequence analysis have been used to characterize and differentiate phytoplasma strains by amplifying the highly conserved gene coding in the dispersed regions of the 16S rRNA [28]. The nested-PCR assay is designed to overcome the low concentration of phytoplasma in infected samples through increasing both sensitivity and specificity [29]. Our research was designed with the following objectives: (i) the detection and molecular identification and characterization of the phytoplasma associated with sesame under our study conditions, (ii) studying the impacts of phytoplasma on the ultrastructural modification of infected sesame plants, and (iii) to determine the impact of phytoplasma infection on agro-physio-biochemical traits, seed and oil yield and their components, and the oil’s fatty acid profile.

## 2. Results

### 2.1. Symptomatology and Phytoplasma Detection

**Figure 1:**
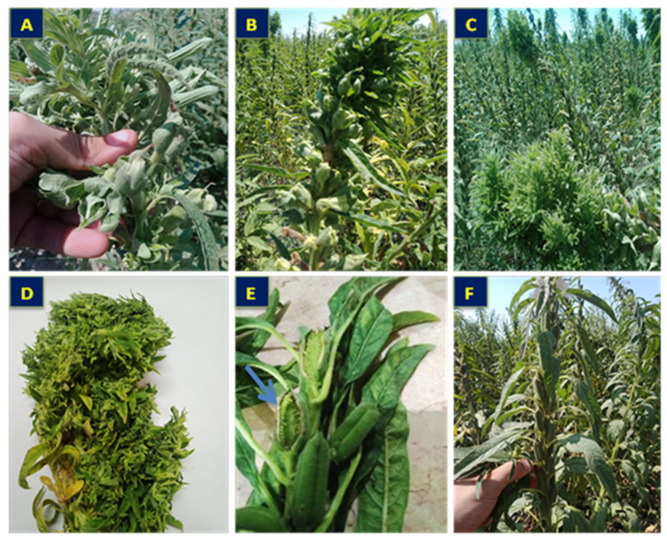
Phyllody symptoms associated with phytoplasma of naturally infected sesame plants: (**A**) phyllody, (**B**) floral proliferation with virescence, (**C**) proliferation of auxiliary shoots, (**D**) yellowing with short internodes and small leaves, (**E**) germinated seeds in cracked capsules, and (**F**) healthy plants.

**Figure 2:**
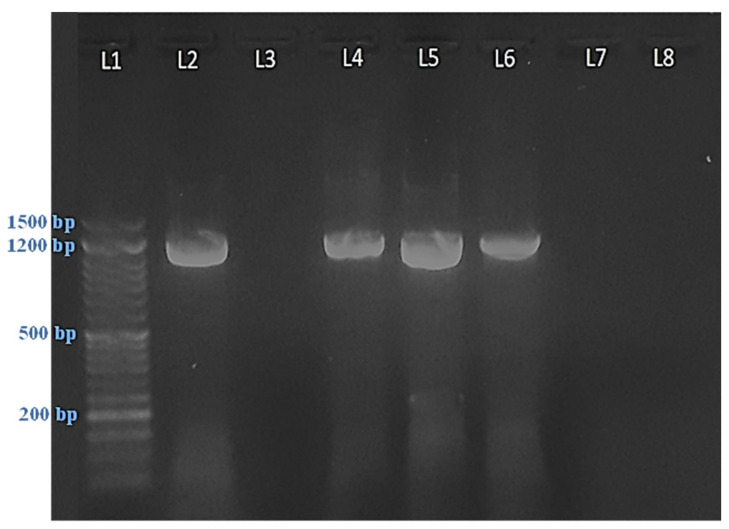
Agarose gel electrophoresis of polymerase chain reaction (PCR) products from the 16S rDNA gene used for the detection of phytoplasma in sesame plants via nested-PCR. L1: 50 bp DNA ladder (M). L2: Positive control sample. L3: Negative control sample. L4, L5, and L6: Symptomatic sesame leaf samples. L7 and L8: Healthy control samples.

The symptomatic samples of sesame plants in our study showed symptoms related to phytoplasmas, such as phyllody (Figure 1A), floral virescence (Figure 1B), the proliferation of auxiliary shoots (Figure 1C), yellowing with short internodes and small leaves (Figure 1D), and the cracking of seed capsules of germinated seeds (Figure 1E), compared to healthy plants (Figure 1F). PCR amplification was successfully used for phytoplasma detection using nested-PCR via the universal primer pairs P1/P7 and R16F2n/R16R2. All symptomatic samples gave positive results with a product size of approximately 1200 bp, while the healthy plants gave no results (Figure 2).

### 2.2. Sequence Analysis

**Figure 3:**
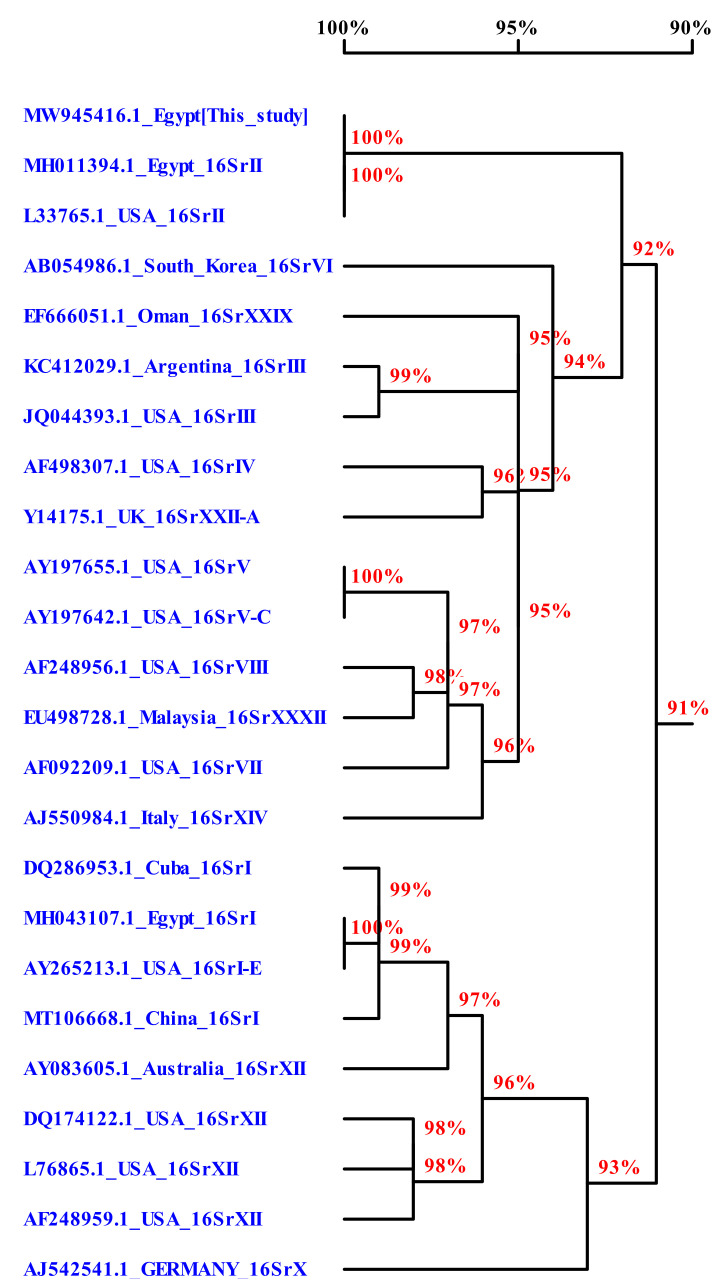
Phylogenetic tree constructed from partial 16S rDNA sequences for Egyptian isolate MW945416 compared to other isolates available in the GenBank database.

Phylogenetic analysis (Figure 3) was performed to compare our Egyptian isolate (deposited in the National Center for Biotechnology Information (NCBI) GenBank under an MW945416 accession number) with the sequences of other phytoplasma strains in the GenBank database (NCBI, Bethesda, MD, USA). The results indicate that our isolate was a member of the group 16SrII, and it very closely (99.7%) identified with isolates of MH011394 and L33765.1 from Egypt and USA, respectively, which belong to the same subgroup (Table S1). In addition, the comparison showed that the Egyptian isolate (MW945416) shared 92.8% and 92.7% sequence homology with the USA (JQ044393.1) and Argentina (KC412029) isolates, respectively, which belong to the 16Sr III group, while the homology with other subgroups ranged between 89.8% and 91.2%, as shown in Table S1.

### 2.3. Histopathological Changes

**Figure 4:**
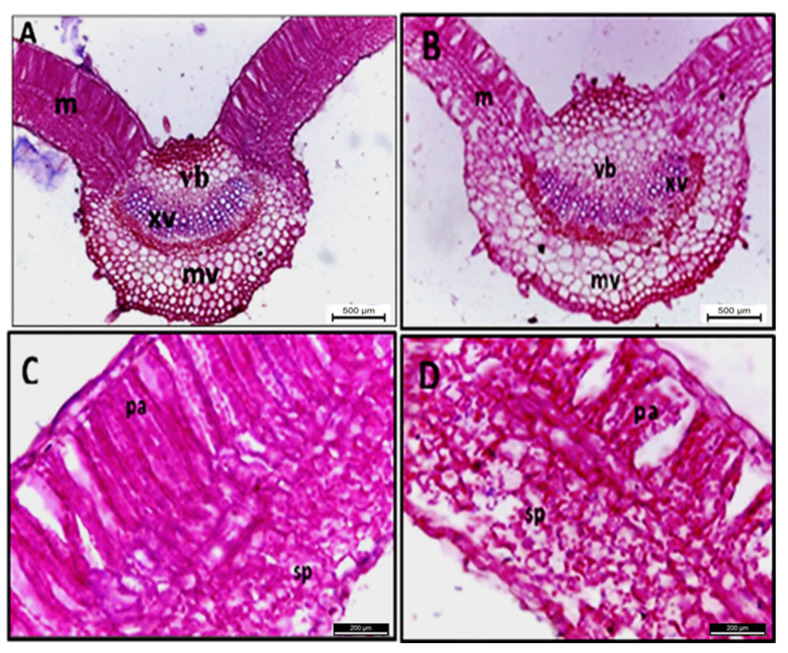
Transections of sesame plant leaf for healthy (**A**,**C**) and phytoplasma-infected (**B**,**D**) plants. m = mesophyll, mv = midvein, pa = palisade tissue, sp = spongy tissue, vb = vascular bundle, and xv = xylem vessels. Scale bar for (**A**,**B**) = 500 µ and (**C**,**D**) = 200 µ.

The anatomical structure of sesame leaves was markedly affected by phytoplasma infection, as presented in Table 1 and Figure 4. This infection reduced the leaf blade’s thickness by 12.2% compared with healthy ones. This reduction resulted from the decrease in both spongy and palisade tissues by 25.0% and 10.3%, respectively. The palisade tissue was irregular, with a wide intercellular space, and lost its normal shape due to the deformation of the cell wall, in addition to the spongy tissue being malformed. Leaf midvein was negatively affected and malformed by phytoplasma infection, whereby its width and length increased by 14.1% and 24.2%, respectively. This increase resulted from the increase in width and length of the vascular bundle by 6.3% and 31.3%, respectively, accompanied by an increase in phloem and xylem zone thickness. Most anatomical changes were observed in the bundle tissue, including the phloem, such as disarrangement and malformation.

**Figure 5:**
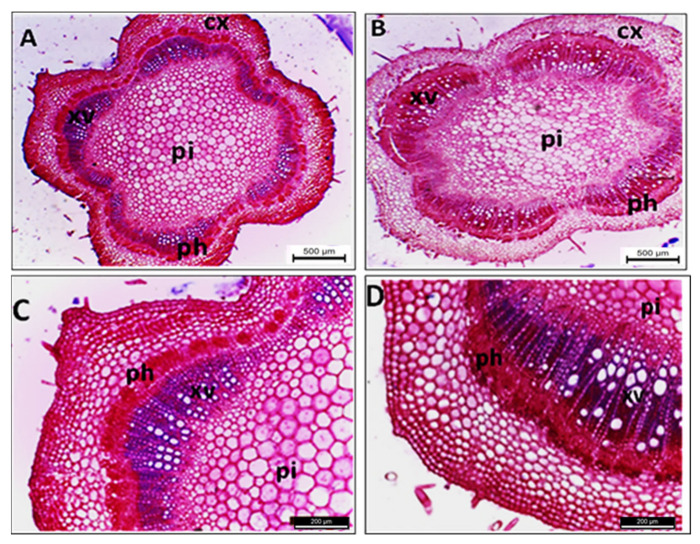
Transections of sesame plant stem for healthy (**A**,**C**) and phytoplasma-infected (**B**,**D**) plants. cx = cortex, vb = vascular bundle, ph = phloem, xv = xylem vessels, and pi = pith. Scale bar for (**A**,**B**) = 500 µ and (**C**,**D**) = 200 µ.

The data presented in Table 2 and Figure 5 show that several anatomical changes occurred in the stems of phytoplasma-infected sesame plants; for example, the stem section diameter was increased by 39.0% compared with the healthy one. This increase was due to the increase in pith diameter by 16.8%. Additionally, all other stem components increased, such as cortex thickness (by 30.4%) due to the increased cortex layer number, and the vascular cylinder thickness increased by 41.1%. In addition, marked increases in vascular tissue thickness (by 254.8%) were recorded because of the increase in phloem and xylem zone thickness by 234.1% and 375.3%, respectively, with malformation and disorganization of the phloem tissue.

**Figure 6:**
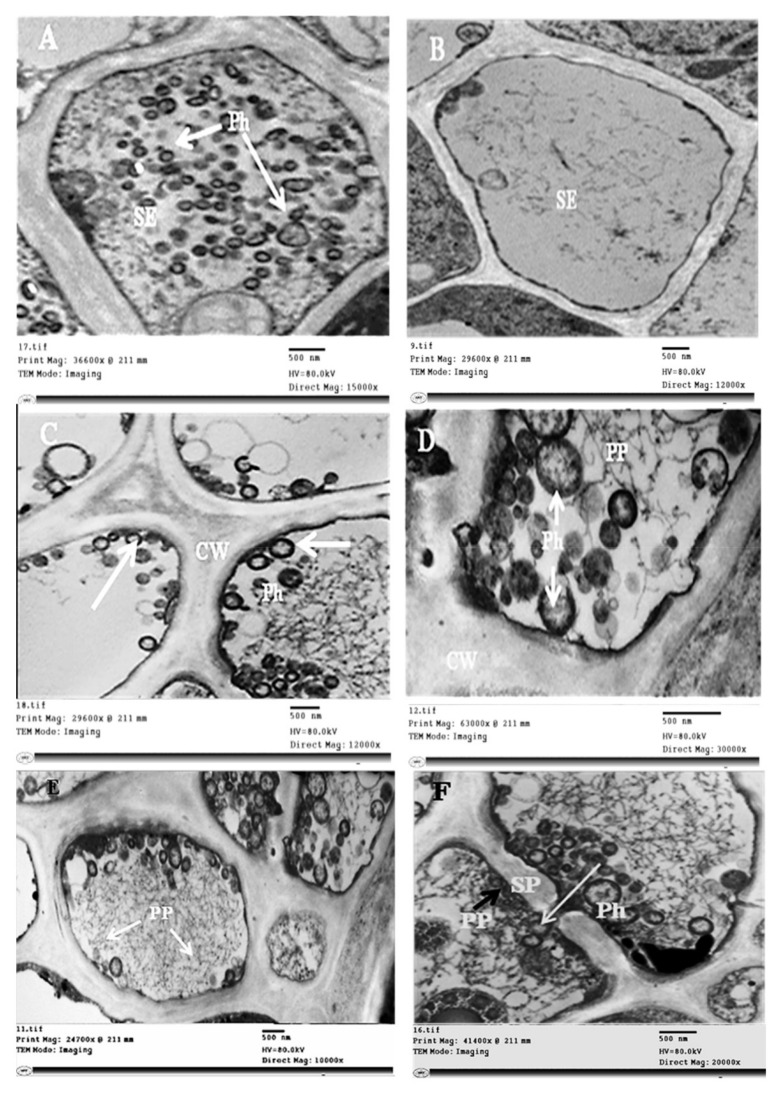
Transmission electron micrographs of the phloem cells from the healthy and infected sesame leaves. (**A**) Spherical or pleomorphic phytoplasma units in the sieve elements of infected phloem tissue (white arrows), (**B**) no phytoplasma units were observed in the sieve elements of healthy plants, (**C**) groups of phytoplasma particles attached to the plasma membrane of the sieve elements (white arrows), (**D**,**E**) simple or branched strands and thin networks of protein filaments (the white arrows) and phytoplasma in the budding stage, and (**F**) dense meshwork of protein filaments surrounding phytoplasmas and sieve plate pores (black arrow), phytoplasma units passing through sieve plate pores (white arrow). SE: sieve elements; ph: phytoplasma; pp: phloem protein; sp: sieve plate.

On the other hand, ultrathin sections of healthy and infected sesame leaves examined by electron microscopy showed the presence of phytoplasma units in the SEs of infected phloem tissue. These units were spherical or pleomorphic, bounded by cell membrane without a cell wall, and measuring 100–600 nm in size (Figure 6A), while no phytoplasma bodies were observed in healthy plants (Figure 6B). Phytoplasma bodies were observed floating separately (Figure 6A) or in groups linked to the plasma membrane of the SEs (Figure 6C). Phytoplasma was noticed in the budding stage, and different organizational forms of protein filaments were observed in SEs. These forms of phloem protein included simple or branched strands forming thin networks (Figure 6D,E), or a dense meshwork surrounding phytoplasmas and sieve plate pores that occluded the sieve pores (Figure 6F).

**Figure 7:**
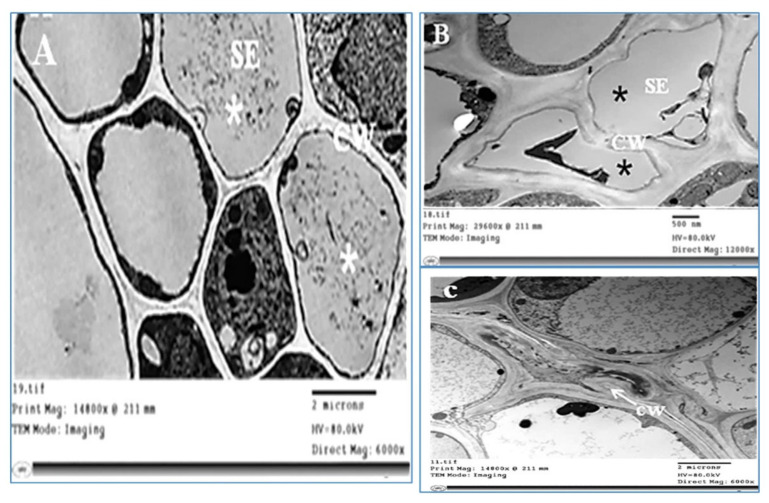
Transmission electron micrographs of the phloem cells from the healthy and infected sesame leaves. (**A**) Phloem cells of the healthy sesame plants (white asterisks), (**B**) malformation and disorganization of phloem cells of infected plants (black asterisks), (**C**) cell wall was thicker and had many dentations (white arrow). CW = cell wall, SE = sieve elements.

**Figure 8:**
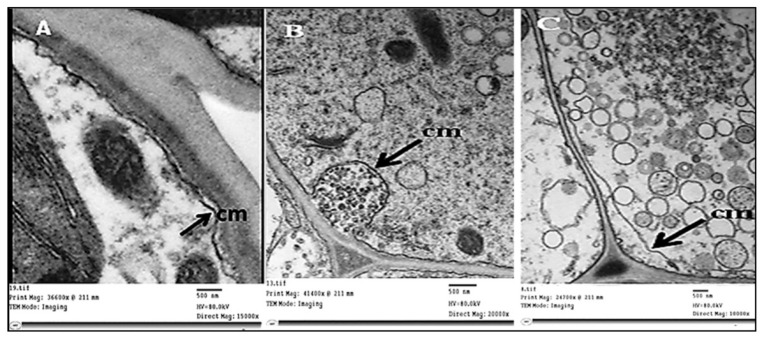
A transmission electron micrograph of ultrathin sections revealed an irregular plasma membrane. (**A**,**B**) The undulating and bulging plasma membrane (black arrows), plasma membrane separated from the cell wall, and phytoplasma attached to it (**C**). cm = cell membrane.

Concerning the ultrastructural changes that occurred in infected plant tissue, our investigations revealed obvious general disorganization, with abnormalities in the cells of the phloem tissue (Figure 7B). Uneven thickness and an irregular shape in the cell wall were obvious (Figure 7C) in phytoplasma-infected plants compared with healthy ones (Figure 7A). The plasma membrane of infected SEs was deformed as a result of undulating, bulging, or separating from the cell wall (Figure 8A, Figure 8B, and Figure 8C, respectively).

**Figure 9:**
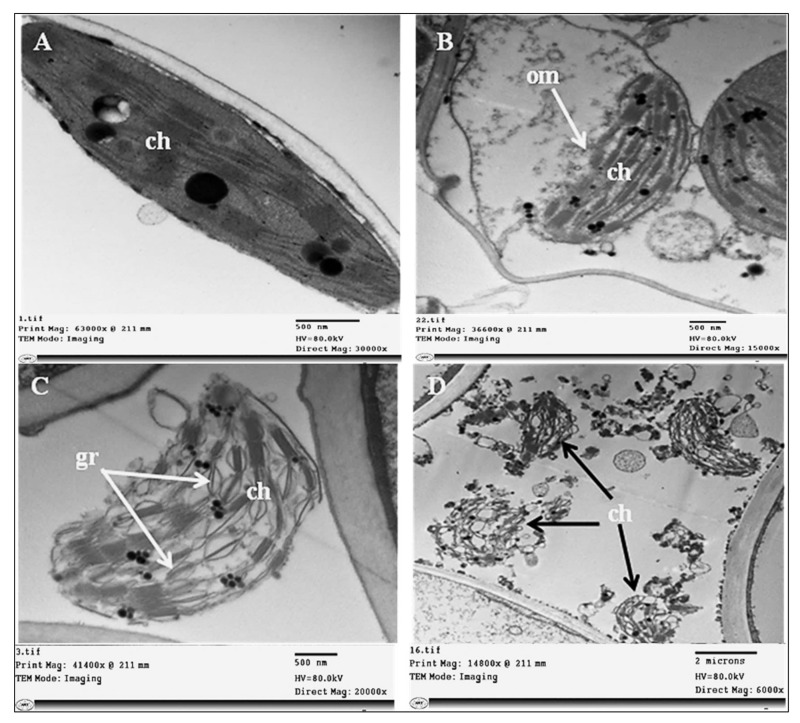
Ultrastructure of damaged chloroplasts of sesame infected by phytoplasma. (**A**) Regular shape of chloroplast of healthy leaf cells, (**B**) degradation and partial lysis in the outer and inner membranes of chloroplasts of infected cells (white arrow), (**C**) complete lysis in membranes and irregular arrangements of grana and thylakoid stacks with signs of destruction in structure (white arrows), and (**D**) all chloroplasts in the infected cell were fully destroyed and broken down (black arrows). Ch = chloroplast, gr = grana, om = outer membrane.

**Figure 10:**
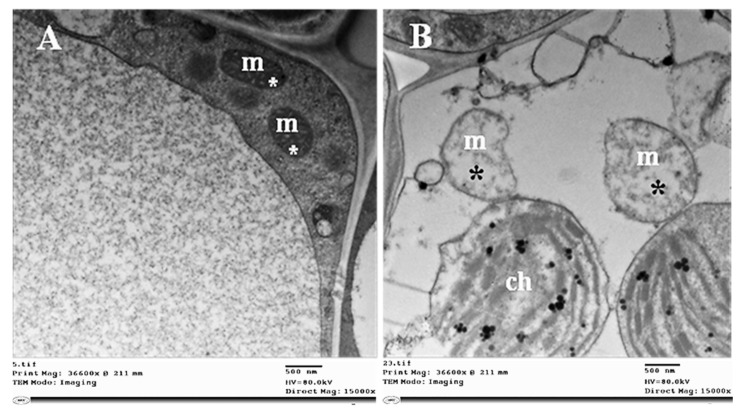
Ultrastructure of the changed mitochondria of sesame infected by phytoplasma. (**A**) Regular shape of mitochondria of healthy leaf cells (white asterisks), and (**B**) degraded or lysed mitochondria due to phytoplasma infection (black asterisks). Ch = chloroplast, and m = mitochondria.

Phytoplasma infection-induced damage in chloroplasts, whereby their outer and inner membranes were partially degraded (Figure 9B), was followed by complete lysis of both membranes. Irregular grana arrangements and thylakoid stacks with signs of destruction in their structures were observed (Figure 9C). Finally, all chloroplasts in some cells were fully destroyed and broken down (Figure 9D). In addition, we observed structural abnormalities in the mitochondria, which became enlarged and swollen, and their internal cristae were disrupted (Figure 10B).

### 2.4. Changes in Phytoplasma-Related Primary and Secondary Metabolites

Compared with healthy sesame plants, phytoplasma-infected plants (*p* ≤ 0.05) exhibited considerably higher (by 29.5%) total soluble proteins as the primary metabolite, and secondary metabolites in the leaf tissues increased too, such as total phenolic content, total flavonoid content, total tannins, and total alkaloids, by 165.9%, 64.7%, 16.7%, and 461.5%, respectively, at the Biologische Bundesanstalt, Bundessortenamt und CHemische Industrie 70 (BBCH 70) phenological stage (Table 3).

### 2.5. Morpho-Physiological Responses in Phytoplasma-Infected Sesame Plants

The sesame’s morpho-physiological attributes responded (*p* ≤ 0.05) dramatically after phytoplasma infection at the beginning of the pods setting (BBCH 70) stage, as shown in Table 4. The main plant responses observed included shortening and thinning of the main stem by 42.1% and 20.9%, respectively, increases in the first-order branch number per plant^−1^ by 46.2%, narrowing in the leaf area per plant^−1^ by 64.5%, reduced weight of the dry plant^−1^ by 41.7%, reduced leaf greenness index by 46.1%, and decreased relative water content in leaf tissues by 11.4%, compared to the healthy sesame plants.

### 2.6. Changes in Productivity, Seed Harvest Index, Seed Protein, and Seed Oil

Compared with healthy sesame plants (Table 5), phytoplasma-infected plants exhibited a similar biological yield, but noticeably lower seed yield plant^−1^, seed index, seed yield, oil yield, seed harvest index, and seed oil content, which were 38.0%, 10.0%, 37.9%, 42.5%, 47.2%, and 7.2% of those of healthy plants. On the other hand, seed protein content increased by 10.1% in phytoplasma-diseased sesame plants compared with healthy plants.

### 2.7. Physio-Chemical Changes in Sesame Oil Properties

The oil extracted from sesame seeds was evaluated to identify its physio-chemical changes due to phytoplasma infection (Table 6). The properties of refractive index, flow rate, acid value, and acidity were statistically similar to those of healthy sesame oil. Meanwhile, the peroxide value in oil from phytoplasma-infected sesame plants was 43.2% higher than that of healthy plants.

### 2.8. Changes in the Fatty Acid Composition of Sesame Oil

**Figure 11:**
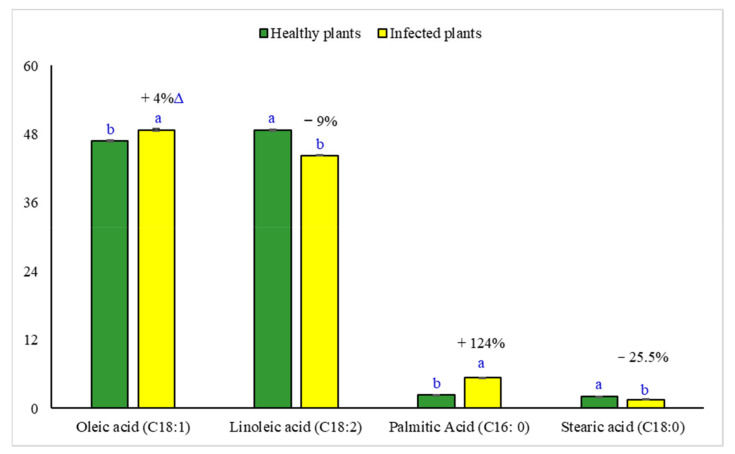
Fatty acid composition (%) of sesame oil produced from healthy and phytoplasma-infected sesame plants grown for the 2021 summer season. Each bar represents mean ± standard error (n = 3 replicates), and each two bars sharing the same letter for each fatty acid are not significantly (*p* ≤ 0.05) different, according to Student’s *t*-test. ^∆^ Value refers to the percentage change due to phytoplasma infection.

The fatty acid profile of sesame oil was determined to explore the extent of the changes in oil quality under phytoplasma infection conditions (Figure 11). Oil from phytoplasma-infected sesame exhibited a level of monounsaturated oleic acid (C18:1) that was higher by 4.0%, and saturated palmitic acid (C16:0) that was lower by 124.4%, compared with oil from healthy plants. However, healthy sesame oil displayed levels of polyunsaturated linoleic acid (C18:2) that were higher by 9.0%, and saturated stearic acid (C18:0) that was lower by 25.5%, compared with oil from phytoplasma-infected plants.

### 2.9. Correlation Analysis

**Figure 12:**
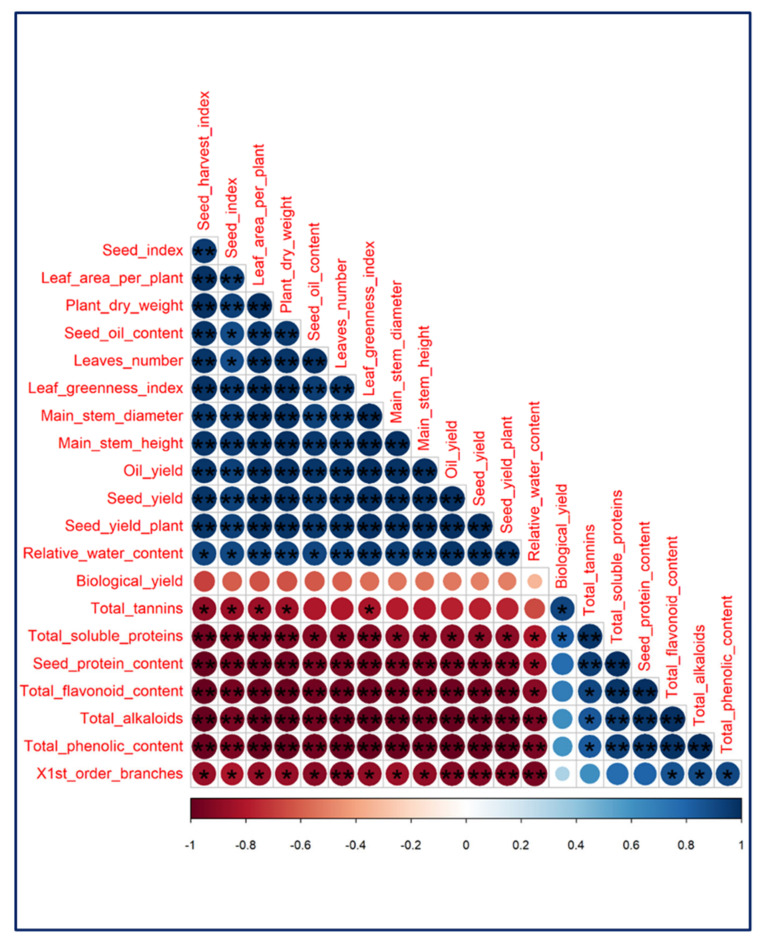
Heatmap of simple Pearson’s correlation coefficients (r) matrix of different sesame parameters in this study, where the colored scale indicates the positive (dark blue) or negative (dark red) correlation, and the “r” coefficient ranges from −1.0 to 1.0. * and ** refer to significant correlations at *p* ≤ 0.05 and *p* ≤ 0.01, respectively.

Bivariate correlation analysis was performed on the data obtained from healthy and phytoplasma-infected plants to explore the relations between the seed or oil yield and other studied traits (Figure 12). The highest significant positive correlations were found between seed or oil yield and seed yield plant^−1^ (0.996 ** or 0.995 **), main stem height (0.994 ** or 0.995 **), leaf greenness index (0.994 ** or 0.992 **), plant dry weight (0.986 ** or 0.987 **), main stem diameter (0.988 ** or 0.984 **), leaf area per plant (0.984 ** or 0.983 **), leaves number plant^−1^ (0.975 ** or 0.979 **), seed harvest index (0.974 ** or 0.978 **), relative water content (0.974 ** or 0.972 **), and seed oil content (0.964 ** or 0.973 **). The highest significant negative correlations were observed between seed or oil yield and total phenolic content (0.993 ** or 0.992 **), total alkaloids content (0.990 ** or 0.990 **), total flavonoid content (0.968 ** or 0.971 **), first-order branch number per plant^−1^ (0.923 ** or 0.927 **), and total soluble proteins (0.896 * or 0.901 *).

## 3. Discussion

Crop plants in arid and semi-arid climates are often exposed to many environmental stresses during their lives, which hinders their yield performance. These stresses can be abiotic, such as drought, salinity, heavy metals, and weeds [30,31,32,33,34], or biotic, such as pests and diseases [35]. Nowadays, phytoplasmas are among the most serious plant pathogens that negatively affect economically valuable crops, such as sesame, threatening worldwide food security [36]. Therefore, developing more phytoplasma-tolerant sesame genotypes is imperative to resolve future phytoplasma-induced threats to worldwide sesame production. However, this demands extensive investigations into and knowledge about the adaptive tolerance mechanisms and responses to phytoplasma infection that support sesame plant survival. Moreover, very little research on phytoplasma-induced alterations in sesame crop has been conducted, and no commercial genotypes resistant to phytoplasma have been developed for use at the world scale.

Our study area, a dry environment (Table 7), has shown epidemic infection with sesame phytoplasma, in addition to low fertility and soil salinity. Our findings reveal that phytoplasma-infected sesame plants exhibit a wide range of symptoms, as mentioned in Figure 1. These symptoms have been described by El-Banna et al. [20] and Youssef et al. [9] in Egypt and other many countries, including Paraguay [37], Turkey [38], Pakistan [19], and India [39].

Historically, phytoplasma diagnosis and phylogenetics have been based on the 16S rRNA gene because of the availability of universal primers for this region [40]. In the current study, nested-PCR with degenerate primer P1/P7, followed by nested primer R16R2/R16F2n, was successfully used for phytoplasma detection in all tested sesame plants, even with low-titer phytoplasma [41]. 16S rDNA is very beneficial for use in the classification of phytoplasma into groups/subgroups, and for determining genetic diversity, which is a necessary step in phytoplasma study [42]. The phytoplasma groups in our study were determined by nucleotide sequencing, and phylogenetic analysis confirmed that our phytoplasma isolates belonged to the 16SrII group because they shared 99.7% sequence homology with the Egyptian isolate from the sesame fields in Sharkia governorate, which belong to this group. Candidatus phytoplasma from the group16SrII was previously found in Egypt on eggplant, tomato, and squash plants by Omar and Foissac [43], on periwinkle, onion, and cactus plants by El-Sisi et al. [44], and on gazania by Gad et al. [45].

Phytoplasma infection leads to various anatomical changes in the stem and leaf tissues of phytoplasma-infected sesame plants compared with healthy ones (Table 2 and Table 3). These changes included irregular spongy and palisade tissues [9], disorganization, and the malformation of vascular bundle tissue. Similar observations have previously been reported by El-Banna and El-Deeb [46], Randall et al. [47], and Ahmed et al. [48].

Electron microscopic examination of the infected sesame plants revealed the presence of pleomorphic phytoplasma units linked to the SE’s plasma membrane, which consume the cell membrane’s sterols to meet their energy needs for growth and division [49,50]. Phytoplasma units were observed at various concentrations in different phloem cells, and this has been related to symptom severity in agreement with Kesumawati et al. [51] and El-Banna et al. [27]. In contrast, Kaminiska et al. [52] reported no correlation between the number of phytoplasma units and the severity of rose phyllody symptoms. We observed different organizations of the protein filaments in SEs surrounding phytoplasmas, as well as of sieve plate pores [53,54]. The SE’s protein filaments play a crucial role in defense mechanisms against phytoplasma infection, and the aggregation of SE protein filaments facilitates a better defensive performance [55]. Both *AtSEOR1* and *AtSEOR2* were reported to be necessary for the formation of SE’s protein filaments [53]. Phytoplasma infection caused various ultrastructural changes, as shown in Figure 7, Figure 8, Figure 9 and Figure 10. These ultrastructural changes resulting from phytoplasma infection may be attributed to the accumulation of sugars and starch, which disturbs sieve tube functionality and disrupts phloem transport [24,27,48]. The phytoplasma infection destroyed the grana and stroma lamellae structure of chloroplasts and impaired their photosynthetic capacity, due to the downregulation of genes that are involved in the photosynthesis process [23]. These unfavorable changes adversely affect the chloroplast’s functionality, causing photosynthetic process inhibition and thus abnormal carbohydrate accumulation in the infected plants. Ultrastructural changes due to phytoplasma infection such as those observed in our study, have been reported by Buxa et al. [56] and Santi et al. [57].

Changes in primary (i.e., soluble proteins) and secondary (i.e., phenolics, flavonoids, tannins, and alkaloids) metabolite levels in response to phytoplasma infection were explored in our study, in order to assess the extent of metabolic adaptation to disease through alterations in the biochemical processes [21,58,59,60]. Our results in Table 4 revealed that phytoplasma infection significantly increased the total soluble proteins and total phenolics. These findings are in line with those of Thangjam and Vastrad [59] and Rasool et al. [60]. The increased protein biosynthesis in infected sesame plants may result from the metabolic activation of the plant host’s natural defense mechanisms against phytoplasma infections [61]. The formation of inbuilt defensive proteins boosts the host plant’s resistance to pathogen invasions [58,62]. Our findings suggest a phytoplasma-induced boost in the levels of polyphenol compounds (e.g., flavonoids, phenolics, tannins, and alkaloids) in host plant tissues. These results are consistent with those reported by Ahmad et al. [21], Junqueira et al. [63], Reveles-Torres et al. [64], and Negro et al. [65]. Polyphenols are well-known antimicrobial compounds involved in plant defense mechanisms, acting as signal molecules to activate plant defense genes, reactive oxygen species (ROS) scavengers, and singlet oxygen quenchers in the host plant [64,66]. A high level of polyphenols in plants after infection reflects the host’s response to phytoplasma infection, and their increased accumulation could be related to the defense mechanisms of the host [63]. The increased levels of polyphenols in phytoplasma-infected sesame leaves in our study may be related to the phytoplasma-induced biosynthesis of L-phenylalanine [67] and hydroxycinnamic acid [65], which boost the phenylpropanoid metabolic pathway, leading to higher levels of various polyphenol compounds. Growth reduction and leaf yellowing in the sesame plants resulted from phytoplasma infection (Table 4), which caused growth impairment, and consequently the hindrance of the uptake, transportation, and utilization of the soil water and ions required for healthy growth [68]. Some of these mineral ions (mainly N, magnesium, and iron) may be necessary for chlorophyll pigment biosynthesis, which may be related to mechanisms supporting the structural and functional integrity of the photosynthetic machinery [69]. As one of the first visible symptoms of disease progression, leaf chlorosis might be exacerbated by poor soil fertility. In this context, Buoso et al. [70] reported that iron deficiency in phytoplasma-infected plants had negative impacts on chlorophyll pigment and young leaf chlorosis in tomato plants. The sesame’s lower leaf greenness index (i.e., chlorophyll content) in our study related to the severe damage of the thylakoid stacks, structural and functional destruction of the grana and stroma lamellae of chloroplasts (Figure 9), and increased numbers of starch granules in the infected plant’s leaves [23]. Another possibility is the increase in chlorophyll degradation via enhancing the activity of the chlorophyllase enzyme under the conditions of phytoplasma infection [71]. Additionally, a lower content of cellular 5-aminolevulinic acid, a key precursor in the chlorophyll biosynthesis route, is another possible explanation for the leaf chlorosis seen in phytoplasma-infected plants [72]. Although the sesame’s photosynthetic rate was not measured in this study, it can be presumed that the reduction in chlorophyll content could impair sesame’s photosynthetic capacity, as mentioned in the pathosystem of coconut lethal phytoplasmic yellowing [24]. The chlorophyll pigment is necessary for the absorption and transmission of incident light energy, as well as photosystem II photoprotection, and its depletion in infected sesame leaves could be among the primary factors affecting the photosynthetic rate [73]. The increasing number of first-order branches in phytoplasma-infected sesame plants (Table 4) may be due to the phytoplasma’s S54LP of the SP gene, which encodes the Aster yellows phytoplasma witch’s broom (AY-WB) effector SAP54 protein that induces sesame phyllody [35]. Further, the imbalanced homeostasis of plant hormones (i.e., zeatin, gibberellin acid (GA), abscisic acid (ABA), and indole-3-acetic acid (IAA)) could be another reason for the increased branch number, concomitant with the broom-like growth of phytoplasma-infected sesame plants [25,74,75]. Youssef et al. [9] found that IAA, GA, and cytokinin abscisic acid increased, but ABA decreased in phytoplasma-infected sesame plant leaves. Once the sesame plants had been subjected to phytoplasma infection in our study, the oil yield drastically dropped because of the lowering of the seed yield and its components and the seed oil content (Table 5). These findings relate to the decreased leaf RWC and chlorophyll content, as well as the limited photosynthetic capacity, and the consequently hindered growth of sesame. The leaves on crop plants are well-known source organs because of their photosynthetic functions; however, the leaves on witch’s broom shoots have limited photosynthetic capacities [25,73]. This could be the main reason for the weakened seed and oil yield and could contribute to the attributes of phytoplasma-infected sesame crop plants. Other reasons could include the flower deformation (abnormal zygomorphic flowers forming instead of normal actinomorphic flowers), the irreversible loss of flower fertility, and the presence of seedless capsules induced by hormonal imbalance and the damage caused by the effectors secreted by pathogenic phytoplasma cells [76]. However, most seeds in diseased plants are small (seed index; Table 5) due to their slow phenological development at the post-blooming stage. This may be due to the severe level of infection at the beginning of the post-blooming stage, resulting from the higher phytoplasmic titer, as reported by Singh et al. [35] in their sesame phyllodic disease progression study. The presence of a higher phytoplasmic titer localized in sink organs could be due to the mobility of the phytoplasma, fed on the phloem sap’s sugar, from source organs (green leaves) toward sink organs (reproductive parts). The decline in sesame seed oil (Table 5) might be attributable to the fact that phytoplasmic infection converts leaves from source to sink organs via an increase in starch accumulation [23], resulting in fewer assimilates being allocated for oil biosynthesis in the seeds. The sesame seeds’ oil and protein contents showed a negative relationship under our study conditions (Table 5). This negative relationship was observed in sesame [77,78] and other oil crops [79]. This inverse relation between oil and protein content may be related to their competition during seed development for the sparse organic carbon skeletons (mainly sugars) that favor protein biosynthesis during energy metabolism [80]. Phytoplasma infection had no notable impact on any of the oil’s physio-chemical properties, except for its peroxide value (Table 6). The peroxide index is an important index illustrating oil oxidation degree and its final quality, and fresh vegetable oil should have a peroxide value of less than 10 meq O_2_ kg^−1^. The existence of peroxides is related to the development of degradative processes, which cause the oil to spoil, leading to flavor, color, and odor changes. There are no existent studies on the physio-chemical properties of the oils from sesame or other crops infected by phytoplasma. The high peroxide value of oil produced from phytoplasma-infected sesame plants may be attributed to the lipid peroxidation resulting from the higher levels of ROS, which increase in response to phytoplasma infection-induced biotic stress [81]. Diseased plants are expected to produce sesame oil with a short shelf-life, as it has a high peroxide value. The main components of sesame oil are unsaturated healthy fatty acids—mainly oleic and linoleic acids—that have potential health benefits for humans [82]. Oleic acid has high stability, good frying properties, and high heat tolerance, but linoleic acid may lower blood cholesterol levels and the likelihood of cardiovascular diseases. Our results show increased oleic and decreased linoleic fatty acids in phytoplasma-infected sesame plants, compared to healthy plants. This trend is partially consistent with Gholinezhad and Darvishzadeh’s [83] results on the fatty acid profile of oil derived from sesame affected by drought as abiotic stress. A major cause of the increase in oleic fatty acid under phytoplasma infection-induced stress is the sesame plant’s earlier maturity, which will manifest a shorter duration of oilseed filling and a shorter period for converting oleic to linoleic fatty acid [84].

## 4. Materials and Methods

### 4.1. Site Description, Experimental Details, and Plant Material

During the summer of 2020, a survey was conducted in some sesame growing regions of Fayoum governorate, Egypt, including our study area, and all phytoplasma-related disease symptoms were observed. Furthermore, our pathogenesis survey results (unpublished data) confirmed that this area was highly infected (epidemic levels) with sesame phytoplasma. So, we selected three separated experimental fields with an area of 1000 ± 150 m^2^ to carry out our main study in the subsequent sesame growing season of 2021. These three experimental fields (29°16′ N, 30°63′ E, and 23 m a.s.l) were located in Al-Prince village, Itsa district, Fayoum province, Egypt. According to the aridity indication [85], the experimental location is characterized by a typical semi-arid agro-climate (Table 7). The soil (0–0.6 m depth) has a sandy loam texture [86], with an electrical conductivity of 3.47 dS m^−1^ in the soil paste extract, a pH of 7.65, total nitrogen (N) content of 0.04 mg kg^−1^, available potassium content of 40.1 mg kg^−1^, available phosphorous content of 3.54 mg kg^−1^, and organic matter content of 0.95 mg kg^−1^, a dry bulk density of 1.44 g cm^−3^, and hydraulic conductivity of 2.41 cm h^−1^.

Twenty experimental units in each experimental field were carefully constructed to conduct this experiment. Each experimental unit consisted of six ridges of 4 m length and 0.6 m width forming a 14.4 m^2^ net area. These experimental units in each field were divided into two different groups, ten for each, the first group intended for healthy sesame plants and the other for natural phytoplasma-infected plants. To maintain a non-infected healthy crop in the first group during the season, the sesame plants were continuously foliar-sprayed with Actara^®^ 25% WG (Syngenta crop protection Inc., Basel, Switzerland, active substance thiamethoxam) at a concentration of 96 g 480^−1^ L ha^−1^ water every 15 days up to BBCH 60 (beginning flowering stage). The other group was left without any defense against the insect vector to encourage natural phytoplasma infection, based on the pathogenesis survey outcomes of this region conducted in the 2020 season. This research was conducted on a *Sesamum indicum* L. cultivar Giza 32 (secured from the Egyptian Agriculture Ministry) that was manually sown on 21 May 2021 and harvested on 10 September of the same year. Healthy and uniform-sized sesame seeds were carefully selected and manually sown using the dry method, 20 cm apart, at a rate of 3 to 5 seeds per hill on one side of a ridge. The sesame seedlings were carefully thinned to maintain two healthy plants per hill at the 4–6 unfolded leaves stage (~21 days from sowing). According to the local practice of commercial sesame crop production, all other advised agronomical practices were followed, such as irrigation and fertilization management and weeding.

### 4.2. Source of Samples

From each experimental unit, fifteen phytoplasma-infected sesame plants and an equal number of non-infected healthy plants (control) were randomly selected and carefully labeled. These selected and labeled plants were assigned to plant sampling groups for the detection and identification of phytoplasma, and other laboratory and field measurements were taken during the growing and at harvest time. One hundred and fifty sesame samples showing phyllody symptoms (Figure 1) and an equal number of asymptomatic samples were collected at BBCH 60 (beginning flowering stage) from each experimental group. The collected leaf samples were first detected by electron microscopy and then confirmed by nested-PCR.

### 4.3. Nucleic Acid Extraction

Total DNA was extracted from the symptomatic and asymptomatic sesame plants collected from Itsa district, Fayoum governorate, Egypt using the modified Dellaporta extraction method [87].

### 4.4. Polymerase Chain Reaction (PCR)

Universal phytoplasma-specific primers were used as described by Casati et al. [88] and Franova et al. [89], as shown in Table 8. For the first PCR, the primer pair P1/P7 was used to amplify a 1.8 kb product of 16s rRNA gene, and the primer sets R16F2n/R16R2 were nested within the primer-annealing positions P1/P7 for the second PCR amplification of 1.2 kb products. A PCR reaction was performed on a 25 µL solution containing 2 μL DNA, 12.5 µL amaR one Master mix (Gene Direx, Inc., Taoyuan, Taiwan), 1.5 μL of 25 pmol of each primer, and 7.5 μL of sterile water.

The DNA was amplified in an applied biosystem DNA Thermal Cycler (Proflex PCR system; Applied Biosystems, Waltham, MA, USA) via 35 cycles of melting, annealing, and DNA extension (1 cycle of 3 min at 94°, 30 cycles of 1 min at 94 °C, 2 min at 53 °C and 55 °C for the nested primer, 2 min at 72 °C, and a final extension of 10 min at 72 °C). The temperature was then reduced to 4 °C and the reaction mixtures were removed. The amplified DNA was electrophoresed in 1% agarose gel in 0.5× Tris-borate-EDTA buffer at 100 V for 1 h, which was stained with EZview nucleic acid stain (Biomatik, Kitchener, ON, Canada).

### 4.5. PCR Cleanup

Samples that gave positive results on the gel were cut and cleaned using the Gel/PCR DNA Fragments Extraction Kit (Geneaid Biotech Ltd., Taipei, Taiwan).

### 4.6. Sequencing and Analysis

The DNA fragments were sent to Macrogen Inc. (Seoul, Korea) for sequencing. The obtained sequences were analyzed and compared to their equivalent GenBank sequences by performing a BLAST comparison using a DNAMAN 7.0 software program (Lynnon BioSoft, Vaudreuil, QC, Canada).

### 4.7. Sampling and Measurements

#### 4.7.1. Histopathological Changes

Light and electron microscopy were carried out to study the anatomical and ultrastructural changes induced in tissue and cell components by phytoplasma infection.

#### 4.7.2. Light Microscopy

The stem and leaf midribs of infected and healthy sesame plants were killed and fixed for at least 48 h in formalin glacial acetic acid, before being dehydrated. They were then serially sectioned by a rotary microtome at 20 μ thickness, and finally double-stained with crystal violet and erythrosine, cleared in carbol xylene, and mounted in Canada balsam [90].

#### 4.7.3. Transmission Electron Microscopy

Electron microscopy was carried out using the tissues of the leaf midribs of infected and healthy sesame. The samples were prepared, fixed, and dehydrated as described by El-Banna et al. [17]. The samples were then sectioned at a thickness of 90 nm using a Leica model EM-UC6 ultra-microtome (LEICA EM-UC6, Leica Microsystems, Wetzlar, Germany) and deposited on thin-bar 400 mesh copper grids. The sections were then double-stained with uranyl acetate and lead citrate (2%; *w*/*v*) and examined via a JEOL JEM-1400 transmission electron microscope (JEOL Ltd., Tokyo, Japan) at the specified magnification. Images were captured using a charge-coupled device optronics camera, camera model AMT, with a 1632 × 1632-pixel format as the side-mount configuration.

#### 4.7.4. Determination of Phytoplasma-Related Primary and Secondary Metabolites

The procedure outlined in Bradford [91] was used to extract and determine the total soluble proteins (mg g^−1^ dry weight; DW) of fully expanded sesame leaf tissues. The total phenolic content of sesame leaves was extracted and estimated following Folin–Ciocalteu’s spectrophotometric method, detailed in Singleton and Rossi [92]. The total phenolic content of the extracted samples was expressed as mg of gallic acid equivalents (GAEs) per gram DW (mg GAEs g^−1^ DW). The total flavonoid content of dry sesame leaves was quantified following the aluminum chloride spectrophotometric method described by Jia et al. [93]. The total flavonoid content of the samples was expressed as mg of quercetin equivalents (QUEs) per g DW of extract (mg QUEs g^−1^ DW). The total tannins content was quantified using the Folin–Ciocalteu reagent and sodium carbonate solution following the spectrophotometric method described by Saxena et al. [94]. The total tannins content of the sesame leaf extract was expressed as mg of tannic acid equivalents (TAEs) per g DW (mg TAEs g^−1^ DW). The total alkaloids content was quantified following the non-spectrophotometric (weighing) method described by Nagpurkar and Patil [95].

#### 4.7.5. Morpho-Physiological Responses

At BBCH 70 (beginning of pods setting), five sesame plants were randomly collected from both healthy and phytoplasma-infected experimental units (n = 150). These plants were promptly transported to the laboratory to measure their morpho-physiological responses, namely, the main stem height (cm) at the meter-scale, 1st-order branch number per plant^−1^, leaves number per plant^−1^, main stem diameter (cm), and leaf area per plant (dm^2^) using a portable digital planometer (Planix 7), for healthy leaves only. The plant dry weight (g) was determined via digital balance after electric oven-drying at 70 ± 2 °C until a constant weight was attained. The sesame leaf greenness index was used to express the relative chlorophyll concentration (Soil–Plant Analysis Development (SPAD) reading) in healthy and phytoplasma-infected plants, measured using a hand-held SPAD-502 chlorophyll meter (Minolta Inc., Tokyo, Japan). Using the uppermost wholly expanded young sesame leaves, the relative water contents in healthy and phytoplasma-infected leaves were measured using the Jones and Turner [96] method.

#### 4.7.6. Yield and Its Components and Seed Harvest Index

At the BBCH 81 ripping stage (capsules turning from light green to yellow), ten sesame plants were harvested from each experimental unit (n = 300) of healthy and phytoplasma-infected sesame plants. These harvested plants were oven-dried at 70 ± 2 °C until a constant weight was reached for biological yield (t ha^−1^) estimation. After that, the sesame seeds were threshed, cleaned, and weighed to determine seed yield plant^−1^ (g) and seed yield (kg ha^−1^). For the estimation of seed yield based on 14% seed moisture, a 200 g subsample was weighed, oven-dried to a constant dry weight at 105 ± 2 °C, and then weighed once more. The seed index (g) was used to express the 1000-seed weight. The seed harvest index, which is the ratio of seed to biological yield (as described by Sinclair [97]), was calculated as follows: Seed harvest index = [seed yield (t ha^−1^)/biological yield (t ha^−1^)] × 100.

#### 4.7.7. Total Seed Protein, Seed Oil Content, and Oil Yield

For seed protein estimation, dried and homogenized samples of sesame seeds were digested in a HClO_4_ and H_2_SO_4_ mixture (1:3, *v*/*v*, respectively) and diluted to 100 mL with deionized water. The total N concentration was estimated as described in Kjeldahl’s method, described in Section 4.2.04 [98]. Sesame seed protein content was calculated by multiplying the total N% by a 6.25 converting factor. According to the Ag 1–65 method [99], the oil from the milled sesame seeds was chemically extracted via a Soxhlet appliance using petroleum ether (b.p. 40–60 °C, secured from Merck Co., Darmstadt, Germany) as an extraction solvent for 8 h. The residual solvent in the extraction samples was eliminated by oven-drying at 60 ± 2 °C for 1 h. The oil content as g per 100 g milled seed was computed by comparing the weights of milled seed samples pre- and post-oil extraction process. The oil yield (kg ha^−1^) was computed as follows: OY (kg ha^−1^) = Oil (%) × SY (kg ha^−1^).

#### 4.7.8. Sesame Oil Extraction and Oil Physio-Chemical Properties Determination

In total, 200 g of healthy and phytoplasma-infected sesame seeds were randomly sampled and cleaned by separating out any foreign material. Oil extraction was performed in triplicate by soaking the crushed seeds in n-hexane and shaking for 24 h at room temperature. The miscella was filtered through no. 1 Whatman filter paper. This filtrated miscella was well mixed, and the oil was retrieved via desolventization using a Buchi R-114 rotary vacuum evaporator at 50 °C. The collected oil was dehydrated over anhydrous Na_2_SO_4_ and then preserved at −20 °C in darkish brown vials to determine the refractive index using a refractometer (Carl-Zeiss, Jena, Germany). The data were standardized at 25 °C [100], and the flow rate was determined using an Ostwald viscometer at 40 °C, according to Joslyn’s [101] method. Acid value (mg KOH g^−1^) and acidity (%) were determined following the Ca 5a-40 [102] and Cd 3d-63 methodologies [103], respectively. The peroxide value as milliequivalents of O_2_ kg^−1^ oil (meq O_2_ kg^−1^ oil) was determined following the Cd 8b-90 methodology [104].

#### 4.7.9. Quantification of Sesame oil Fatty Acid Composition

The fatty acid methyl esters (FAMEs) derivatized from sesame oil were analyzed using GC-MS apparatus (Agilent Technologies Inc., Santa Clara, CA, USA). A different FAMEs content, as a percentage of total fatty acids, was identified by comparing our mass spectra data with those from the Wiley and NIST mass spectral library.

#### 4.7.10. Statistical Analysis

The data on each parameter were subjected to Levene’s homogeneity testing [105] and Shapiro–Wilk’s normality test [106] before analysis. The outputs show that both the homogeneity and normality tests gave good results for parametric statistics and simple correlation analyses. The significant differences between the healthy and phytoplasma-infected groups were determined using the two-tailed Student’s *t*-test with IBM^®^ SPSS^®^ (SPSS Inc., IBM Corporation, Armonk, NY, USA, version 25) for Windows. A probability level ≤ 0.05 was considered statistically significant in all tests that we ran.

## 5. Conclusions

In conclusion, phylogenetic analysis has confirmed the applicability of phytoplasma detection via nested-PCR assays, and we have classified our Egyptian isolate (with the accession number MW945416) as a member of the 16SrII group. Various anatomical and ultrastructural changes occurred in the tissue and cellular components of phytoplasma-infected sesame plants. Phytoplasma infection led to remarkable increases in the assessed primary (i.e., total soluble proteins) and secondary (i.e., phenolics, flavonoids, tannins, and alkaloids) metabolites of infected sesame plants. Phytoplasma infection also caused a decrease in plant water status and chlorophyll pigment, as well as growth impairment, considerable seed and oil yield loss, and the deterioration of the oil’s physio-chemical properties. Our study findings represent a significant step toward a better understanding of the phytoplasma–host plant interaction mechanism, and they may be beneficial in overcoming phytoplasma infection. We recommend further research to produce phytoplasma-resistant sesame genotypes to protect them from future threats of phytoplasma infection and improve their productivity. This can be realized by better understanding their adaptive mechanisms and response to phytoplasma infection.

## Figures and Tables

**Table 1:** Leaf anatomical structure of the healthy and phytoplasma-infected sesame plants at the beginning of flowering (Biologische Bundesanstalt, Bundessortenamt und CHemische Industrie 60 (BBCH 60) stage), grown during the 2021 summer season.

Parameters (μm)	Healthy Sesame	Infected Sesame	Change (%) ^∆^
Upper epidermis thickness	13.74	10.42	−24.2
Lower epidermis thickness	14.59	9.82	−32.7
Midvein width	757.13	864.00	+14.1
Midvein length	569.86	707.56	+24.2
Blade thickness	256.84	225.39	−12.2
Palisade tissue thickness	103.02	77.26	−25.0
Spongy tissue thickness	135.65	121.64	−10.3
Vascular bundle width	516.70	549.22	+6.3
Vascular bundle length	135.04	177.35	+31.3
Protoxylem vessels height	14.06	18.94	+34.7
Protoxylem vessels width	18.48	21.26	+15.1
Metaxylem vessels height	24.21	23.59	−2.5
Metaxylem vessels width	27.43	26.20	−4.5

^∆^ Percentage change compared with those of healthy plants.

**Table 2:** Stem anatomical structure of the healthy and phytoplasma-infected sesame plants at the beginning of flowering (BBCH 60 stage) grown during the 2021 summer season.

Parameters (μm)	Healthy Sesame	Infected Sesame	Change (%) ^∆^
Stem diameter	2788.56	3876.16	+39.0
Cortex thickness	217.34	283.38	+30.4
Number of cortex layers	10.00	12.00	+20.0
Vascular cylinder thickness	2260.59	3190.25	+41.1
Vascular tissues thickness	339.71	1205.23	+254.8
Phloem zone thickness	79.33	265.09	+234.1
Xylem zone thickness	228.87	1087.74	+375.3
Pith diameter	1487.40	1737.42	+16.8

^∆^ Percentage change compared with those of healthy plants.

**Table 3:** Phytoplasma-related primary and secondary metabolites of healthy and phytoplasma-infected sesame plants at the beginning of pods setting (BBCH 70 stage) grown during the 2021 summer season.

Parameter	Healthy Plants	Infected Plants	Change (%) ^∆^	*p*-Value
Total soluble proteins (mg g^−1^ DW)	2.24 ± 0.04 b	2.90 ± 0.12 a	+29.5	0.005 **
Total phenolic content (mg GAEs g^−1^ DW)	5.02 ± 0.04 b	13.35 ± 0.09 a	+165.9	<0.001 **
Total flavonoid content (mg QUEs g^−1^ DW)	0.99 ± 0.01 b	1.63 ± 0.05 a	+64.7	<0.001 **
Total tannins (mg TAEs g^−1^ DW)	0.24 ± 0.01 b	0.28 ± 0.01 a	+16.7	0.036 *
Total alkaloids (mg g^−1^ DW)	1.04 ± 0.02 b	5.84 ± 0.04 a	+461.5	<0.001 **

The values are means ± standard error (n = 3 replicates). Each two means for each parameter in each row sharing the same letter do not differ significantly at *p* ≤ 0.05, according to Student’s *t*-test (* and ** point to differences at *p* ≤ 0.05, and *p* ≤ 0.01, respectively). ^∆^ Percentage change compared with those of healthy plants.

**Table 4:** Morpho-physiological responses of healthy and phytoplasma-infected sesame plants at the beginning of pods setting (BBCH 70 stage) grown during the 2021 summer season.

Parameter	Healthy Plants	Infected Plants	Change (%) ^∆^	*p*-Value
Main stem height (cm)	180.33 ± 6.1 a	104.33 ± 2.3 b	−42.1	<0.001 **
1st-order branches number plant^−1^	4.50 ± 0.5 b	6.58 ± 0.2 a	+46.2	0.014 *
Leaves number plant^−1^	109.00 ± 2.6 a	70.43 ± 2.3 b	−35.4	<0.001 **
Main stem diameter (cm)	1.63 ± 0.03 a	1.29 ± 0.02 b	−20.9	<0.001 **
Leaf area per plant (dm^2^)	67.92 ± 1.6 a	24.09 ± 0.1 b	−64.5	0.001 **
Plant dry weight (g)	114.21 ± 2.0 a	66.63 ± 1.8 b	−41.7	<0.001 **
Leaf greenness index	48.51 ± 0.9 a	26.17 ± 0.7 b	−46.1	<0.001 **
Relative water content (%)	77.43 ± 1.2 a	68.60 ± 1.0 b	−11.4	0.004 **

The values are means ± standard error (n = 3 replicates with 50 plants in each). Each two means for each parameter in each row sharing the same letter do not differ significantly at *p* ≤ 0.05, according to Student’s *t*-test (* and ** point to differences at *p* ≤ 0.05, and *p* ≤ 0.01, respectively). ^∆^ Percentage change compared with those of healthy plants.

**Table 5:** Yield and its components, seed harvest index, total seed protein, and seed oil content of healthy and phytoplasma-infected sesame plants grown during the 2021 summer season.

Parameter	Healthy Plants	Infected Plants	Change (%) ^∆^	*p*-Value
Seed yield plant^−1^ (g)	17.61 ± 0.35 a	10.91 ± 0.29 b	−38.0	<0.001 **
Seed index (g)	3.61 ± 0.05 a	3.25 ± 0.04 b	−10.0	0.005 **
Seed yield (kg ha^−1^)	1467.0 ± 26.5 a	910.4 ± 24.0 b	−37.9	<0.001 **
Oil yield (kg ha^−1^)	756.2 ± 16.9 a	435.2 ± 10.9 b	−42.5	<0.001 **
Biological yield (t ha^−1^)	6.18 ± 0.37 a	7.30 ± 0.65 a	+18.1	0.206 ^ns^
Seed harvest index	23.95 ± 0.20 a	12.65 ± 0.78 b	−47.2	<0.001 **
Seed protein content (%)	19.32 ± 0.10 b	21.27 ± 0.24 a	+10.1	0.002 **
Seed oil content (%)	51.54 ± 0.28 a	47.81 ± 0.40 b	−7.2	0.002 **

The values are means ± standard error (n = 3 replicates with 100 plants in each). Each two means for each parameter in each row sharing the same letter do not differ significantly at *p* ≤ 0.05, according to Student’s *t*-test (* and ** point to differences at *p* ≤ 0.05, and *p* ≤ 0.01, respectively, ^ns^ = not significant at *p* ≤ 0.05). ^∆^ Percentage change compared with those of healthy plants.

**Table 6:** Physio-chemical oil properties of healthy and phytoplasma-infected sesame plants grown during the 2021 summer season.

Parameter	Healthy Plants	Infected Plants	Change (%) ^∆^	*p*-Value
Refractive index	1.474 ± 0.00 a	1.474 ± 0.00 a	0.00	>0.9999 ^ns^
Flow rate	5.43 ± 0.02 a	5.47 ± 0.06 a	+0.74	0.500 ^ns^
Acid value (mg KOH g^−1^)	3.18 ± 0.21 a	3.28 ± 0.61 a	+3.14	0.887 ^ns^
Acidity (%)	1.60 ± 0.10 a	1.65 ± 0.31 a	+3.12	0.887 ^ns^
Peroxide value (meq O_2_ kg^−1^ oil)	9.57 ± 0.53 b	13.70 ± 0.63 a	+43.2	0.007 **

The values are means ± standard error (n = 3 replicates). Each two means for each parameter in each row sharing the same letter do not differ significantly at *p* ≤ 0.05, according to Student’s *t*-test (** points to differences at *p* ≤ 0.01 and ^ns^ = not significant at *p* ≤ 0.05). ^∆^ Percentage change compared with those of healthy plants.

**Table 7:** Monthly averages of some agro-climatic parameters in the experimental area, Egypt, during the 2020 growing summer season.

Month	Temperature (°C)		Relative Humidity (%)	Wind Speed (m s^−1^)	Precipitation (mm)	Solar Radiation(MJ m^−2^ d^−1^)
	Day	Night				
May	34.2	17.2	36.1	3.73	0.00	28.7
June	36.9	19.9	31.9	3.70	0.30	31.6
July	38.2	22.0	34.4	3.68	0.00	32.7
August	38.7	22.6	36.2	3.42	0.00	30.7
September	38.1	22.4	40.3	3.74	0.00	28.3

**Table 8:** Primer sequences and the size of the amplified PCR product.

Primer	Sequence	Size of the PCR Product
P1	AAGAGTTTGATCCTGGCTCAGGATT	1.8 kb
P7	CGTCCTTCATCGGCTCTT	
R16F2n	GAAACGACTGCTAAGACTGG	1.2 kb
R16R2	TGACGGGCGGTGTGTACAAACCCCG	

## Data Availability

Data will be made available by the authors upon written request.

## Supplementary Materials

The following supporting information can be downloaded at: https://www.mdpi.com/article/10.3390/plants11040477/s1, Table S1: Identity the sequence of phytoplasma under study and other available on the GenBank.

## References

[1] Kabak B., Dobson A.D. (2017). Mycotoxins in spices and herbs—An update. Crit. Rev. Food Sci. Nutr..

[2] Jayaraj P., Narasimhulu C.A., Rajagopalan S., Parthasarathy S., Desikan R. (2020). Sesamol: A powerful functional food ingredient from sesame oil for cardioprotection. Food Funct..

[3] Uzun B., Arslan C., Furat S. (2008). Variation in fatty acid composition, oil content and oil yield in a germplasm collection of sesame (*Sesamum indicum* L.). J. Am. Oil Chem. Soc..

[4] Rizki H., Nabloussi A., Kzaiber F., Jbilou M., Latrache H., Hanine H. (2018). Comparative assessment of bioactive components, antioxidant effects from 15 cultivars of sesame (*Sesamum indicum* L.) for different crop years. Int. J. Sci. Eng. Res..

[5] Nzikou J.M., Matos L., Kalou G.B., Ndangui C.B., Pambou-Tobi N.P.G., Kimbonguila A., Silou T., Linder M., Desobry S. (2009). Chemical composition on the seeds and oil of sesame (*Sesamum indicum* L.) grown in Congo-Brazzaville. Adv. J. Food Sci. Technol..

[6] Food and Agriculture Organization of the United Nations (FAO) (2020). FAOSTAT Statistics.

[7] MALR (2020). Bulletin of Agricultural Statistics, Arab Republic of Egypt.

[8] FAS (2017). Foreign Agriculture Service/USDA.

[9] Youssef S.A., Safwat G., Shalaby A.B.A., El-Beltagi H.S. (2018). Effect of phytoplasma infection on plant hormones, enzymes and their role in infected sesame. Fresenius Environ. Bull..

[10] Akhtar K.P., Dickinson M., Sarwar G., Jamil F.F., Haq M.A. (2008). First report on the association of a 16SrII phytoplasma with sesame phyllody in Pakistan. Plant Pathol..

[11] Nagaraju, Muniyappa V., Saharan G.S., Mehta N., Sangwan M.S. (2005). Viral and phytoplasma diseases of sesame. Diseases of Oilseed Crops.

[12] Akhtar K.P., Sarwar G., Sarwar N., Elahi M.T. (2013). Field evaluation of sesame germplasm against sesame phyllody disease. Pak. J. Bot..

[13] Salehi M., Esmailzadeh-Hosseini S.A., Salehi E., Bertaccini A. (2017). Genetic diversity and vector transmission of phytoplasmas associated with sesame phyllody in Iran. Folia Microbiol..

[14] Vasudeva R.S., Sahambi H.S. (1955). Phyllody in sesamum (*Sesamum orientale* L.). Indian Phytopathol..

[15] Turkmenoglu Z., Ari U. (1959). A disease-phyllody virus noted on sesame in the Aegean region. Plant Prot. Bull..

[16] Caglayan K., Gazel M., Škorić D., Bertaccini A., Weintraub P., Rao G., Mori N. (2019). Transmission of phytoplasmas by agronomic practices. Phytoplasmas: Plant Pathogenic Bacteria—II.

[17] El-Banna O.M., Mikhail M.S., Farag A.G., Mohamed A.M.S. (2007). Detection of phytoplasma in tomato and pepper plants by electron microscope and molecular biology-based methods. Egypt. J. Virol..

[18] Ahmed E.A., Shalaby O.Y., Dwidar E.F., Mokbel S.A., El-Attar A.K. (2014). Occurrence, etiology and molecular characterization of phytoplasma diseases on *Solanum lycopersicum* crop in Egypt. Egypt. J. Virol..

[19] Akhtar K.P., Dickinson M., Sarwar G., Mushtak A. (2009). Sesame phyllody disease: Its symptomatology, etiology and transmission in Pakistan. Turk. J. Agric. For..

[20] El-Banna O.M., Mikhail M.S., El-Attar A.K., Aljamali A.A.R. (2013). Molecular and electron microscope evidence for an association of phytoplasma with sesame phyllody in Egypt. Egypt. J. Phytopathol..

[21] Ahmad S.J., Farid N., Ahmad J.N. (2019). Metabolic and physiological changes induced in *Sesamum indicum* infected by phytoplasmas. Phytopathog. Mollicutes.

[22] Singh A., Verma P., Lakhanpaul S. (2020). Exploring the methylation status of selected flowering genes in healthy and phyllody infected sesame plants. Phytopathog. Mollicutes.

[23] Xue C., Liu Z., Dai L., Bu J., Liu M., Zhao Z., Jiang Z., Gao W., Zhao J. (2018). Changing host photosynthetic, carbohydrate, and energy metabolisms play important roles in Phytoplasma infection. Phytopathology.

[24] Maust B.E., Espadas F., Talavera C., Aguilar M., Santamaría J.M., Oropeza C. (2003). Changes in carbohydrate metabolism in coconut palms infected with the lethal yellowing phytoplasma. Phytopathology.

[25] Tan Y., Wei H.R., Wang J.W., Zong X.J., Zhu D.Z., Liu Q.Z. (2015). Phytoplasmas change the source-sink relationship of field-grown sweet cherry by disturbing leaf function. Physiol. Mol. Plant Pathol..

[26] Devonshire B.J., Dickinson M., Hodgetts J. (2013). Visualization of phytoplasmas using electron microscopy. Phytoplasma. Methods in Molecular Biology (Methods and Protocols).

[27] El-Banna O.M., Toima N.I., Youssef S.A., Shalaby A. (2015). Molecular and electron microscope evidence for an association of phytoplasma with Citrus Witches Broom disease. Int. J. Sci. Eng. Res..

[28] Youssef S.A., Sayed Y., Hassan O.S., Safwat G., Shalaby A. (2017). Universal and specific 16S-23Sr RNA PCR primers for identification of phytoplasma associated with sesame in Egypt. Int. J. Adv. Res. Biol. Sci..

[29] Gundersen D.E., Lee I.M., Rehner S.A., Davis R.E., Kingsbury D.T. (1994). Phylogeny of mycoplasma like organisms (phytoplasmas): A basis for their classification. J. Bacteriol..

[30] Mekdad A.A.A., Shaaban A., Rady M.M., Ali E.F., Hassan F.A.S. (2021). Integrated application of K and Zn as an avenue to promote sugar beet yield, industrial sugar quality, and K-use efficiency in a salty and semi-arid agro-ecosystem. Agronomy.

[31] Shaaban A., Al-Elwany O.A.A.I., Abdou N.M., Hemida K.A., El-Sherif A.M.A., Abdel-Razek M.A., Semida W.M., Mohamed G.F., Abd El-Mageed T.A. (2022). Filter Mud Enhanced Yield and Soil Properties of Water-Stressed *Lupinus termis* L. in Saline Calcareous Soil. J. Soil Sci. Plant Nutr..

[32] Mekdad A.A.A., El-Sherif A.M.A., Rady M.M., Shaaban A. (2021). Culture management and application of humic acid in favor of *Helianthus annuus* L. oil yield and nutritional homeostasis in a dry environment. J. Soil Sci. Plant Nutr..

[33] Abd El-Mageed T.A., Rady M.O., Semida W.M., Shaaban A., Mekdad A.A. (2021). Exogenous micronutrients modulate morpho-physiological attributes, yield, and sugar quality in two salt-stressed sugar beet cultivars. J. Soil Sci. Plant Nutr..

[34] Mekdad A.A., El-Enin M.M.A., Rady M.M., Hassan F.A., Ali E.F., Shaaban A. (2021). Impact of level of nitrogen fertilization and critical period for weed control in peanut (*Arachis hypogaea* L.). Agronomy.

[35] Singh V., Kumar S., Lakhanpaul S. (2018). Differential distribution of phytoplasma during phyllody progression in sesame (*Sesamum indicum* L.) under field conditions—An important consideration for effective sampling of diseased tissue. Crop Prot..

[36] Hemmati C., Nikooei M., Al-Subhi A.M., Al-Sadi A.M. (2021). History and current status of phytoplasma diseases in the Middle East. Biology.

[37] Junior E.D.J.G., Segnana L.G., Kitajima E.W., Bedendo I.P. (2019). Sesame phyllody associated with a 16SrI-B phytoplasma, a ‘Candidatus Phytoplasma asteris’-related strain, in Paraguay. Sci. Agric..

[38] Baspinar H., Korkmaz S., Onelge N., Cinar A., Uygun N., Kersting U. (1993). Studies on citrus stubborn disease pathogen and sesame phyllody in sesame and their related leafhopper vector. J. Turk. Phytopathol..

[39] Venkataravanappa V., Reddy C.N.L., Manjunath M., Chauhan N.S., Reddy M.K. (2017). Detection, characterization and in-silico analysis of candidatus phytoplasma australasia associated with phyllody disease of sesame. Adv. Plants Agric. Res..

[40] Smart C.D., Schneider B., Blomquist C.L., Guerra L.J., Harrison N.A., Ahrens U., Lorenz K.H., Seemüller E., Kirkpatrick B.C. (1996). Phytoplasma-specific PCR primers based on sequences of the 16S-23S rRNA spacer region. Appl. Environ. Microbiol..

[41] Marzachì C. (2004). Molecular diagnosis of phytoplasmas. Phytopathol. Mediterr..

[42] Kakizawa S., Yoneda Y. (2015). The role of genome sequencing phytoplasma research. Phytopathog. Mollicutes.

[43] Omar A.F., Foissac X. (2012). Occurrence and incidence of phytoplasmas of the 16SrII-D subgroup on solanaceous and cucurbit crop in Egypt. Eur. J. Plant Pathol..

[44] El-Sisi Y., Omar A.F., Sidaros S.A., ElSharkawy M.M. (2017). Characterization of 16SrII-D subgroup associated phytoplasmas in new host plants in Egypt. Arch. Phytopathol. Plant Prot..

[45] Gad S.M., Kheder A.A., Awad M.A. (2019). Detection and molecular identification of phytoplasma associated with Gazania in Egypt. J. Virol. Sci..

[46] El-Banna O.M., El-Deeb S.H. (2007). Phytoplasma associated with mango malformation disease in Egypt. J. Phytopathol..

[47] Randall J.J., Bosland P.W., Hanson S.F. (2011). Brote grande, a new phytoplasma associated diseases of chile peppers in the desert southwest. Plant Health Prog..

[48] Ahmed E.A., Shalaby O.Y., Dwidar E.F., Mokbel S.A., El-Attar A.K. (2016). Ultrastructural changes in tomato plant induced by phytoplasma infection and attempts for its elimination using tissue culture techniques. Egypt. J. Virol..

[49] Christensen N.M., Axelsen K.B., Nicolaisen M., Schulz A. (2005). Phytoplasmas and their interactions with hosts. Trends Plant Sci..

[50] Mou H.Q., Lu J., Zhu S.F., Lin C.L., Tian G.Z., Xu X., Zhao W.J. (2013). Transcriptomic analysis of paulownia infected by paulownia witches’ broom phytoplasma. PLoS ONE.

[51] Kesumawati E., Kimata T., Uemachi T., Hosokawa M., Yazawa S. (2006). Correlation of phytoplasma concentration in hydrangea macrophylla with green-flowering stability. Sci. Hortic..

[52] Kaminiska M., Sliwa H., Rudzinska L., Angwald A. (2001). The association of phytoplasma with stunting, leaf necrosis and witches-broom symptoms in magnolia plants. J. Phytopathol..

[53] Anstead J., Froelich D., Knoblauch M., Thompson G. (2012). Arabidopsis P-protein filament formation requires both AtSEOR1 an AtSEOR2. Plant Cell Physiol..

[54] Pagliari L., Buoso S., Santi S., Furch A.C.U., Martini M., Degola F., Loschi A., van Bel A.J.E., Musetti R. (2017). Filamentous sieve element proteins are able to limit phloem mass flow but not phytoplasma spread. J. Exp. Bot..

[55] Bernardini C., Pagliari L., de Rosa V., Almeida-Trapp M., Santi S., Martini M., Buoso S., Loschi A., Loi N., Chiesa F. (2020). Pre-symptomatic modified phytohormone profile is associated with lower phytoplasma titres in an Arabidopsis seor1ko line. Sci. Rep..

[56] Buxa S.V., Degola F., Polizzotto R., Marco F.D., Loschi A., Kogel K.H., Musetti R. (2015). Phytoplasma infection in tomato is associated with re-organization of plasma membrane, ER stacks, and actin filaments in sieve elements. Front Plant Sci..

[57] Santi S., de Marco F., Polizzotto R., Grisan S., Musetti R. (2013). Recovery from stolbur disease in grapevine involves changes in sugar transport and metabolism. Front. Plant Sci..

[58] Hameed S., Akhtar K.P., Hameed A., Gulzar T., Kiran S., Yousaf S., Abbas G., Asghar M.J., Sarwar N. (2017). Biochemical changes in the leaves of mungbean (*Vigna radiata*) plants infected by phytoplasma. Turk. J. Biochem..

[59] Thangjam R., Vastrad A.S. (2018). Biochemical analysis of phytoplasma infected sesame plant transmitted by *Orosius albicinctus* distant. J. Entomol. Zool. Stud..

[60] Rasool A., Jahan M.S., Shazad U., Tariq A., Calica P.N. (2020). Effect of phytoplasma infection on primary and secondary metabolites and antioxidative enzyme activities of sweet orange (*Citrus sinenses* L.). J. Plant Pathol. Microbiol..

[61] Agrios G.N. (1997). Plant Pathology.

[62] Tornero P., Chao R., Luthin W., Goff S., Dangl J. (2002). Large-scale structure, function, analysis, of Arabidopsis RPM1 disease resistance protein. Plant Cell..

[63] Junqueira A., Bedendo I., Pascholati S. (2004). Biochemical changes in corn plants infected by the maize bushy stunt phytoplasma. Physiol. Mol. Plant Pathol..

[64] Reveles-Torres L.R., Velásquez-Valle R., Salas-Muñoz S., Mauricio-Castillo J.A., Esqueda-Dávila K.C.J., Herrera M.D. (2018). Candidatus phytoplasma trifolii (16Sr VI) infection modifies the polyphenols concentration in pepper (*Capsicum annuum*) plant tissues. J. Phytopathol..

[65] Negro C., Sabella E., Nicolì F., Pierro R., Materazzi A., Panattoni A., Aprile A., Nutricati E., Vergine M., Miceli A. (2020). Biochemical changes in leaves of *vitis vinifera* cv. Sangiovese infected by Bois Noir phytoplasma. Pathogens.

[66] Nicholson R.L., Hammerschmidt R. (1992). Phenolic compounds and their role in disease resistance. Ann. Rev. Phytopathol..

[67] Tan Y., Li Q., Zhao Y., Wei H., Wang J., Baker C.J., Liu Q., Wei W. (2021). Integration of metabolomics and existing omics data reveals new insights into phytoplasma-induced metabolic reprogramming in host plants. PLoS ONE.

[68] De Oliveira E., Magalhães P.C., Gomide R.L., Vasconcelos C.A., Souza I.R.P., Oliveira C.M., Cruz I., Schaffert R.E. (2002). Growth and nutrition of mollicute-infected maize. Plant Dis..

[69] Broadley M., Brown P., Çakmak I., Rengel Z., Zhao F., Marschner P. (2012). Function of nutrients: Micronutrients. Marschner’s Mineral Nutrition of Higher Plants.

[70] Buoso S., Pagliari L., Musetti R., Martini M., Marroni F., Schmidt W., Santi S. (2019). Candidatus Phytoplasma solani’ interferes with the distribution and uptake of iron in tomato. BMC Genom..

[71] Bertamini M., Nedunchezhian N., Tomasi F., Grando S. (2002). Phytoplasma [Stolbur subgroup (Bois Noir-BN)] infection inhibits photosynthetic pigments, ribulose-1, 5-biphosphate carboxylase and photosynthetic activities in field grown grapevine (*Vitis vinifera* L. cv. Chardonnay) leaves. Physiol. Mol. Plant Pathol..

[72] Wu Y., Jin X., Liao W., Hu L., Dawuda M.M., Zhao X., Tang Z., Gong T., Yu J. (2018). 5-Aminolevulinic acid (ALA) alleviated salinity stress in cucumber seedlings by enhancing chlorophyll synthesis pathway. Front. Plant Sci..

[73] Ji X., Gai Y., Zheng C., Mu Z. (2009). Comparative proteomic analysis provides new insights into mulberry dwarf responses in mulberry (*Morus alba* L.). Proteomics.

[74] Ding Y., Wei W., Wu W., Davis R.E., Jiang Y., Lee I.M., Hammond R.W., Shen L., Sheng J.P., Zhao Y. (2013). Role of gibberellic acid in tomato defense against potato purple top phytoplasma infection. Ann. Appl. Biol..

[75] Fan G., Dong Y., Deng M., Zhao Z., Niu S., Xu E. (2014). Plant-pathogen interaction, circadian rhythm, and hormone-related gene expression provide indicators of phytoplasma infection in *Paulownia fortunei*. Int. J. Mol. Sci..

[76] Ustun R., Yol E., Ikten C., Catal M., Uzun B. (2017). Screening, selection and real-time qPCR validation for phytoplasma resistance in sesame (*Sesamum indicum* L.). Euphytica.

[77] Li C., Miao H., Wei L., Zhang T., Han X., Zhang H. (2014). Association mapping of seed oil and protein content in *Sesamum indicum* L. using SSR markers. PLoS ONE.

[78] Bellaloui N., Abbas H.K., Ebelhar M.W., Mengistu A., Mulvaney M.J., Accinelli C., Shier W.T. (2018). Effect of increased nitrogen application rates and environment on protein, oil, fatty acids, and minerals in sesame (*Sesamum indicum*) seed grown under Mississippi Delta conditions. Food Nutr. Sci..

[79] Hossain Z., Johnson E.N., Wang L., Blackshaw R.E., Gan Y. (2019). Comparative analysis of oil and protein content and seed yield of five Brassicaceae oilseeds on the Canadian prairie. Ind. Crops Prod..

[80] Kambhampati S., Aznar-Moreno J.A., Hostetler C., Caso T., Bailey S.R., Hubbard A.H., Durrett T.P., Allen D.K. (2020). On the inverse correlation of protein and oil: Examining the effects of altered central carbon metabolism on seed composition using soybean fast neutron mutants. Metabolites.

[81] Xue C., Liu Z., Wang L., Li H., Gao W., Liu M., Zhao Z., Zhao J. (2020). The antioxidant defense system in Chinese jujube is triggered to cope with phytoplasma invasion. Tree Physiol..

[82] Aslam F., Iqbal S., Nasir M., Anjum A.A. (2019). White sesame seed oil mitigates blood glucose level, reduces oxidative stress, and improves biomarkers of hepatic and renal function in participants with type 2 diabetes mellitus. J. Am. Coll. Nutr..

[83] Gholinezhad E., Darvishzadeh R. (2021). Influence of arbuscular mycorrhiza fungi and drought stress on fatty acids profile of sesame (*Sesamum indicum* L.). Field Crops Res..

[84] Nazari M., Mirlohi A., Majidi M.M. (2017). Effects of drought stress on oil characteristics of *Carthamus* Species. J. Am. Oil Chem. Soc..

[85] Ponce V., Pandey R., Ercan S. (2000). Characterization of drought across climatic spectrum. J. Hydrol. Eng..

[86] Soil Survey Staff USDA (1999). Soil Taxonomy. A Basic System of Soil Classification for Making Sand Interpreting Soil Surveys.

[87] Dellaporta S.L., Wood J., Hicks J.B. (1983). A plant DNA minipreparation: Version II. Plant Mol. Biol. Rep..

[88] Casati P., Quaglino F., Tedeschi R., Spiga F.M., Alma A., Spadone P., Bianco P.A. (2010). Identification and molecular characterisation of ‘Candidatus Phytoplasma mali’ isolates in North-Western Italy. J. Phytopathol..

[89] Franova J., Ludvíková H., Paprstein F. (2011). Detection and characterization of phytoplasmas infecting apple trees in Czech Republic during 2010. J. Bull. Insectol..

[90] Nassar M., El-Sahhar K. (1998). Botanical Preparations and Microscopy (Microtechnique).

[91] Bradford M.M. (1976). A rapid and sensitive method for the quantitation of microgram quantities of protein utilizing the principle of protein-dye binding. Anal. Biochem..

[92] Singleton V.L., Rossi J.A. (1965). Colorimetry of total phenolics with phosphomolybdic-phosphotungstic acid reagents. Am. J. Enol. Vitic..

[93] Jia Z.S., Tang M.C., Wu J.M. (1999). The determination of flavonoid contents in mulberry and their scavenging effects on superoxide radicals. Food Chem..

[94] Saxena M., Saxena J., Nema R., Singh D., Gupta A. (2013). Phytochemistry of medicinal plants. J. Pharmacogn. Phytochem..

[95] Nagpurkar M., Patil N.M. (2020). Qualitative and Quantitative Phytochemical Studies in Different Parts of *Sesamum indicum* L. Medicinal Plants: Biodiversity, Sustainable Utilization and Conservation.

[96] Jones M.M., Turner N.C. (1978). Osmotic adjustment in leaves of sorghum in response to water deficits. Plant Physiol..

[97] Sinclair T.R. (1998). Historical changes in harvest index and crop nitrogen accumulation. Crop Sci..

[98] AOAC (1995). Official Methods of Analysis of International, Method.

[99] AOCS (1993). American Oil Chemist’s Society, Method Ag 1–65.

[100] AOAC (2005). Official Methods of Analysis of International.

[101] Joslyn M.A. (1950). Methods in Food Analysis Applied to Plant Products.

[102] AOCS (1998). American Oil Chemist’s Society, Method Ca 5a-40.

[103] AOCS (1999). American Oil Chemist’s Society, Method Cd 3d-63.

[104] AOCS (2011). American Oil Chemist’s Society, Method Cd 8b-90.

[105] Levene H., Olkin I., Ghurye S.G., Hoeffding W., Madow W.G., Mann H.B. (1960). Robust tests of equality of variances. Contributions to Probability and Statistics, Essays in Honor of Harold Hoteling.

[106] Razali N., Wah Y.B. (2011). Power comparisons of Shapiro-Wilk, Kolmogorov-Smirnov, Lilliefors and Anderson-Darling tests. J. Stat. Modeling Anal..

