# Supplementary material for: Effect of Phytoplasma Associated with Sesame Phyllody on Ultrastructural Modification, Physio-Biochemical Traits, Productivity and Oil Quality
Source: Plants (Basel). 2022 Feb 10;11(4):477. doi: 10.3390/plants11040477 (PMC8879811; doi:10.3390/plants11040477)
Supplement: Supplementary file 1 [file plants-11-00477-s001.zip › plants-1587000-supplementary.pdf]

plants

MDPI

**Table S1.** Identity the sequence of phytoplasma under study and other available on the GenBank.

| Accession number on GenBank | Country | 16Sr group and subgroup | Sequence homology |
|---|---|---|---|
| MW945416.1 | Egypt (This study) | | 100.0% |
| MH011394.1 | Egypt | II | 99.7% |
| L33765.1 | USA | II | 99.7% |
| EF666051.1 | Oman | XXIX | 91.5% |
| DQ174122.1 | USA | XII | 90.6% |
| MH043107.1 | Egypt | I | 90.4% |
| KC412029.1 | Argentina | III | 92.7% |
| AF248959.1 | USA | XII | 89.8% |
| AY265213.1 | USA | I-E | 90.4% |
| AF498307.1 | USA | IV | 91.8% |
| AY197655.1 | USA | V | 90.5% |
| AF092209.1 | USA | VII | 91.0% |
| AF248956.1 | USA | VIII | 91.4% |
| AJ542541.1 | Germany | X | 90.3% |
| AJ550984.1 | Italy | XIV | 91.5% |
| Y14175.1 | UK | XXII-A | 92.1% |
| JQ044393.1_ | USA_ | III | 92.8% |
| AY083605.1 | Australia | XII | 90.3% |
| MT106668.1 | China | I | 90.6% |
| L76865.1 | USA | XII | 90.4% |
| AY197642.1 | USA | V-C | 90.4% |
| EU498728.1 | Malaysia | XXXII | 91.2% |
